# Mortality Rate and Related Risk Factors in Hospitalized Coronavirus Disease 2019 Patients with Diabetes: A Single-Center Study: Mortality of COVID-19 and Its Risk Factors in DM Patients

**DOI:** 10.31661/gmj.v11i.2590

**Published:** 2022-12-25

**Authors:** Atousa Najmaldin, Ali Gohari, Hoda Aryan

**Affiliations:** ^1^ Department of Internal Medicine, Semnan University of Medical Sciences, Semnan. Iran; ^2^ Department of Infectious Diseases Medicine, Semnan University of Medical Sciences, Semnan. Iran

**Keywords:** Coronavisus-19, Diabetes Mellitus, Mortality, Hypertension, Cardiovascular Diseases

## Abstract

**Background:**

The outbreak of the new coronavirus disease 2019 (COVID-19) is majorly threatening the health of people worldwide. Since patients with chronic diseases, including diabetes mellitus (DM), are among the main groups at risk of severe COVID-19; hence, this study was aimed to investigate the mortality rate of COVID-19 among patients with DM.

**Materials and Methods:**

This cross-sectional study was performed on 211 DM patients with COVID-19 who were referred to Educational Kowsar Hospital in Semnan, Iran. After a definitive diagnosis of COVID-19, basic characteristics, including gender, weight, height, and clinical information (such as initial signs and symptoms, underlying diseases, complications during hospitalization, and type of treatment received) were collected.

**Results:**

The mean age of patients was 64.92±12.7 years, and 51.7% were male. Totally 20.9% of patients were expired. The most frequent underlying diseases were hypertension and ischemic heart disease. The simultaneous presence of cardiovascular diseases in DM patients with COVID-19 was correlated with a considerable mortality rate increment. Cough on arrival significantly predicted mortality reduction to less than one-third (P=0.009). Also, oxygen saturation of less than 90% on arrival was a significant predictor of an increase in mortality by more than double (P0.001).

**Conclusion:**

According to the results of multivariate logistic regression, it was found that DM can increment the probability of contracting COVID-19, and the rate of mortality was also higher in these patients.

## Introduction

The Coronavirus disease 2019 (COVID-19) has been considered a pandemic disease and has created critical conditions worldwide [1]. At present, the COVID-19 pandemic has become one of the most important health issues in the whole world. Special care is very important for patients with chronic diseases, including hypertension (HTN), diabetes mellitus (DM), cardiovascular diseases (CVDs), as well as the elderly [2]. Also, the mortality rate due to COVID-19 is high in individuals with chronic diseases [3]. The mortality rate of COVID-19 among individuals with DM is 7.3% to 9.2%, whereas this rate is reported to be 1.4% in healthy people [3][4]. Indeed, in the cases of COVID-19, patients with DM experience more severe symptoms and complications. While among patients with well-managed DM, the risk of severe signs and symptoms of COVID-19 is the same as in healthy individuals [3]. In addition, in patients with DM, the frequency of deep vein thrombosis, the rate of thromboembolism, and stroke are several times higher compared to healthy individuals [5][6]. Regarding the increase of DM among Iranian populations [7] and the importance of COVID-19 management in patients with chronic disease, this study aimed to determine the COVID-19 mortality rate in hospitalized patients with DM in Semnan city, Iran.

## Materials and Methods

### Patients

This cross-sectional study was performed on subjects aged 18 years and older with suspected signs and symptoms of COVID-19 who were referred to Kowsar Educational, Research, and Therapeutic Center affiliated with Semnan University of Medical Sciences in Semnan, Iran, from April 2021 to May 2022. All COVID-19 patients included in this study were hospitalized and were diagnosed according to the World Health Organization (WHO) interim guidance [8]. The cases infected with SARS-CoV-2 were confirmed by reverse transcription-polymerase chain reaction (PCR) assay on throat and nose swab samples. Also, chest computed tomography (CT) scan findings were considered for the diagnosis of COVID-19 in the patients with the initial negative PCR test result.

Among 364 patients with a definitive diagnosis of COVID-19, 211 patients with DM were included. Patients with incomplete medical records, missing data, and who left the hospital due to refused medical care were excluded from the study.

### Data Collection

The baseline data, including age, gender, height and weight, COVID-19 confirmation method, clinical signs and symptoms at admission, laboratory findings, chest x-ray and/or CT scans, underlying comorbidities, type of treatments for DM, the need for hospitalization in the intensive care unit (ICU), duration of hospitalization, and the type of treatment received (including antibiotics, antivirals, corticosteroids, oxygen therapy, and mechanical ventilation), and survival status were obtained using standardized data collection forms.

Survival status was defined as the time interval between hospital admission and discharge (survived or deceased). Survivors were defined as patients who were alive at the time of discharge from the hospital. Mortality was measured based on the number of expired patients in all evaluated patients.

### Ethical Considerations

The entry of the participants in the current study was voluntary and informed written consent was acquired from the patients prior to their entry into the research. The participation of patients in the study did not lead to their exclusion from standard treatment and did not involve additional costs for them. Also, the Ethics Committee of Semnan University of Medical Sciences approved the study (approval ethics code: IR.SEMUMS.REC.1399.246).

### Data Analysis

The quantitative data were presented as mean and standard deviation (SD). Also, qualitative data were reported as frequency and percentage. All the analyses were performed with Statistical SPSS Software version 16 (SPSS Inc., Chicago IL, USA) using Chi-square, student t-test, and Mann-Whitney U test. Also, logistic regression was applied to investigate the role of different factors on the mortality rate. The statistically significant differences were considered as P-value less than 0.05.

## Results

**Table:** Table1. Baseline Characteristics of Patients Based Outcome

**Variables**	**Outcomes**		**P-value**
	**Survived**	**Deceased**	
**Age,y (mean±SD)**	62.65±12±67	73.52±9.11	0.01
**BMI, Kg/m ^2^ (mean±SD) **	28.25±5.34	29.37±5.72	0.23
**Gender, n(%)**			
Female	85(83.3)	17(16.7)	0.17
Male	82(75.2)	27(24.8)	
**Smoker**	27(16.2)	13(29.5)	0.04
**Opium consumption**	17(10.2)	7(15.9)	0.29

**BMI:** Body mass index

**Table:** Table2. Initial Presentations of Studied Patients

**Presentations**	*Outcomes		**P-value**	**OR**	**95% CI**		**P-value**
	**Survived**	**Deceased**			**Upper**	**Lower**	
**Cough**	101(60.5)	19(43.2)	0.03	0.301	0.743	0.122	0.009
**Fever**	70(41.9)	20(45.5)	0.39	1.279	3.122	0.524	0.589
**Dyspnea**	63(37.7)	25(56.8)	0.01	1.15	2.763	0.479	0.755
**Myalgia**	60(35.9)	10(22.7)	0.6	0.441	1.198	0.162	0.108
**Headache**	39(23.4)	5(11.4)	0.58	0.549	1.927	0.156	0.349
**Nausea**	32(19.2)	5(11.4)	0.16	0.385	1.786	0.083	0.223
**Vomiting**	24(14.4)	5(11.4)	0.4	1.995	0.068	0.439	0.371
**Anorexia**	29(17.4)	8(18.2)	0.52	1.468	4.68	0.46	0.517
**Decreased sense of smell**	16(9.6)	4(9.1)	0.59	0.752	4.73	0.12	0.761
**Decreased sense of taste**	7(4.2)	3(6.8)	0.34	1.05	9.157	0.12	0.965
**Reduction of oxygen saturation (less than 90)**	31(18.6)	31(70.5)	0.003	7.659	21.164	2.772	<0.001
**Tachycardia**	9(5.4)	15(34.1)	0.464	0.768	7.583	0.412	0.443
**Tachypnea**	8(4.8)	18(40.9)	0.34	2.691	12.79	0.566	0.213

^*^ Data presented as n(%) **OR:** Odds ratio; **CI:** Confidence interval

**Table:** Table3. Frequency of Underlying Diseases Among the Studied Patients

**Presentations**	*Outcomes		**P-value**	**OR**	**95% CI**		**P-value**
	**Survived**	**Deceased**			**Lower**	**Upper**	
**HTN**	98(58.7)	29(65.9)	0.244	0.91	0.42	2	0.82
**IHD**	45(26.9)	24(54.5)	0.001	3.09	1.41	6.74	0.005
**ESRD**	6(30)	3(6.8)	0.282	3.06	0.75	17.9	0.1
**Malignancies**	3(1.8)	3(6.8)	0.107	4.73	0.73	30.49	0.1
**CKD**	18(10.8)	9(20.5)	0.077	1.55	0.57	4.16	0.38
**Pituitary disorder**	2(1.2)	1(2.3)	0.506	6.99	0.42	11.65	0.17
**COPD**	7(4.2)	3(6.8)	0.347	1.43	0.29	7.06	0.65
**Chronic liver disease**	2(1.2)	1(2.3)	0.506	2.15	0.15	29.34	0.56
**Hypothyroidism**	11(6.6)	2(4.5)	0.466	0.25	0.03	1.91	0.18
**Hyperthyroidism**	3(1.8)	2(4.5)	0.279	5.18	0.77	34.56	0.08
**Stroke**	8(4.8)	8(18.2)	0.007	3.91	1.26	12.12	0.01

^*^ Data presented as n(%) **HTN:** Hypertension; **IHD:** Ischemic heart disease; **ESRD:** End-stage renal disease; **CKD:** Chronic kidney disease; C** OPD:** Chronic obstructive pulmonary disease; **OR:** Odds ratio; **CI:** Confidence interval

**Table:** Table4. Frequency Distributions of Different Treatments Methods in Studied Patients

**Type of treatments**	*Outcomes		**P-value**
	**Survived**	**Deceased**	
**Antiviral**	148(89.2)	44(100)	0.012
**Plasmapheresis**	22(13.2)	17(38.6)	0.001
**Corticosteroids**	118(70.7)	39(88.6)	0.01
**Oxygen therapy**	166(99.4)	44(100)	0.791
**NIV**	39(23.4)	40(90.9)	0.001
**Invasive mechanical ventilation **	14(8.4)	43(97.7)	0.001
**Antibiotics**	148(89.2)	44(100)	0.009

^*^ Data presented as n(%) **NIV:** Non-invasive ventilation

**Table:** Table5. Complications of COVID-19 Among Studied Patients

**Presentations**	*Outcomes		**P-value**	**OR**	**95% CI**		**P-value**
	**Survived**	**Deceased**			**Lower**	**Upper**	
**Shock**	2(1.2)	22(47.7)	˂0.001	69.67	10.35	468.78	˂0.001
**AKI**	14(8.4)	19(43.2)	˂0.001	9.54	2.15	42.24	0.003
**Superinfection**	14(8.4)	14(31.8)	˂0.001	4.38	1.08	17.78	0.039
**Arrhythmia**	11(6.6)	13(29.5)	˂0.001	3.32	0.56	19.44	0.183
**ARDS**	8(4.8)	24(54.5)	˂0.001	15.79	3.31	75.25	˂0.001

^*^ Data presented as n(%) **AKI:** Acute kidney injury; **ARDS:** Acute respiratory distress syndrome

**Table:** Table6. Laboratory Findings of DM Patients with COVID-19. Data Presented AS Mean±SD.

**Parameters**	**Outcomes**		**95% CI**		**P-value**
	**Survived**	**Deceased**			**Lower**	**Higher**	
**WBC**	7152.9±6014	10889.5±5602	-5718.1	-1755.09	<0.001
**Hemoglobin**	11.61±2	10.81±1.9	0.12	1.48	0.021
**ESR**	41.1±27.5	62.9±36.8	-33.62	-9.79	0.001
**Ferritin**	324.1±246.2	572.8±272	-332.83	-164.6	<0.001
**BUN**	27.6±24.2	41.4±22.5	-21.78	-5.8	0.001
**CRP**	24.7±18.3	54.4±33.9	-40.36	-19.01	<0.001
**BS**	199.3±88.1	274±127.4	-115.57	-33.8	0.001
**Sodium**	136.8±10.4	140±5.5	-6.43	0.03	0.052
**Potassium**	4.4±0.6	4.6±0.9	-0.56	0.05	0.11
**Creatinine**	1.5±1.5	2.3±3.4	-1.84	0.29	0.151
**Magnesium**	2±0.2	2.1±0.2	-0.0186	-0.007	0.033
**CPK**	170.4±153.9	347.8±484.7	-36.6	-28.39	0.021
**CKMB**	19.8±5.5	24.7±9.7	-8.03	-1.91	0.002
**Troponin**	0.3±0.2	0.3±0.1	-0.105	0.019	0.177
**LDH**	477.7±195	764.1±278.6	-375.87	-196.91	<0.001

**WBC:** White blood cell; **ESR:** Erythrocyte sedimentation rate; **BUN:** Blood urea; **CRP:** C-reactive protein; **BS:** Blood sugar; **CPK:** Creatine kinase; **CKMB:** Creatine kinase-MB; **LDH:** Lactate dehydrogenase

**Table:** Table7. The Role of Some Characteristics of Patients on Mortality

**Variables**	**OR**	**95%CI**		**P-value**
		**Lower**	**Upper**	
**Age more than 61 years **	3.68	1.74	7.77	0.001
**Gender**	1.64	0.83	3.24	0.15
**BMI overweight**	1.6	1.01	2.53	0.042
**Smoker**	2.06	0.9	4.71	0.08
**Opium consumptions**	1.2	0.42	3.38	0.73

**BMI:** Body mass index; **OR:** Odds ratio; **CI:** Confidence interval

### Baseline Characteristics

In this research, among 211 subjects, 109 (51.7%) patients were men, and the mean age of patients was 64.92±12.7 years. The mean body mass index (BMI) was 28.48±5.4 kg/m^2^, and most (41.2%) were overweight (BMI=25-29.9). The mean age of deceased patients was significantly higher than survived patients (P=0.01, Table-1). Results showed that most (63.5%) patients were 61 years old and older, and most had a BMI in the overweight range. However, there were no significant differences between the deceased patients in terms of BMI and age compared to survived patients (Table-1). In addition, it was shown that the mortality was higher (24.8%) in men, but this difference was not considerable (P=0.17, Table-1). Forty (19%) patients were smokers, and 24 (11.4%) patients were addicted to opium (Table-1).Diagnosis of COVID-19 in patients was made via PCR test in three patients, chest CT scan in only one patient, and among the remaining patients, both PCR test and chest CT scan were used.

Also, the most (56.9%) reported symptom was cough (Table-2). The mean duration of hospitalization was 7.39±0.51 days, and 44 (20.9%) patients were died from COVID-19. Regarding underlying diseases (Table-3), HTN was observed in 127 (60.2%) patients, and acute kidney injury (AKI) was reported as the most common (15.6%) complication of COVID-19 (Table-4). Also, oxygen therapy was the most (99.5%) treatment for COVID-19 (Table-5), and 147 (70.1%) patients received oral agents for controlling DM. Laboratory findings of patients are presented in Tabel-6. Laboratory parameters, including ferritin, white blood cell (WBC), erythrocyte sedimentation rate (ESR), blood urea (BUN), C-reactive protein (CRP), blood sugar (BS), creatine kinase (CKP), creatine kinase-MB (CK-MB), lactate dehydrogenase (LDH), and magnesium were significantly higher in deceased patients, and hemoglobin level was significantly lower than in recovered patients. Also, others laboratory parameters, including sodium, potassium, creatinine, and troponin levels, were increased in the deceased patients, but this increase was not significant compared to the levels of these parameters in the recovered patients (Table-6).

In the following, the role of risk factors on the mortality rate of COVID-19 among patients with DM were evaluated using logistic regression.

### The Role of Age, Gender, Habitual, and BMI on the Mortality Rate

As the showed in the Table-7, logistic regression revealed that the mortality increased with increasing age, and by considering the cut-off point of 61 years of age, it was indicated that the rate of increase in mortality was higher than in patients younger than 61 years (odds ratio [OR]: 5.68, 95% confidence interval [CI]: 1.74 to 7.77). Also, increased BMI was correlated with an increase in mortality among patients in the overweight group compared to patients with normal BMI (OR: 1.6, 95%CI: 1.01 to 2.53, P=0.042, Table-7). However, gender, smoking, and opium consumption were not associated with mortality (Table-7).

### Initial Presentation of COVID-19 as the Predictor of Mortality Rate

The initial presentations of patients are mentioned in Table-2. Couth was the most presentation among survived patients. In comparison, oxygen saturation below 90% was observed in 70.5% of deceased patients (Table-2). Regarding logistic regression, the presence of cough, myalgia, headache, nausea, vomiting, and decreased sense of smell was associated with decreased mortality rate. However, fever, dyspnea, anorexia, decreased sense of taste, oxygen saturation of less than 90%, tachycardia, and tachypnea were the predictors of increased mortality (Table-2).

### Underlying Diseases Increased Mortality

HTN was the most common underlying disease in both survived and deceased patients (Table-3). Although the frequency of ischemic heart disease (IHD) and stroke were different among studies patients (P=0.001 and P=0.007, respectively), there were no any significant differences among survived and deceased patients in the term of underlying diseases (P˃0.05, Table-3). Indeed, IHD (OR: 0.91, 95%CI: 1.41 to 6.74, P=0.005) and stroke (OR: 3.91, 95%CI: 1.26 to 12.12, P=0.01) significantly increased mortality among the studied patients (Table-3).

### Effects of Different Treatment Methods on Mortality Rate

Regarding Table-4, oxygen therapy was performed in 99.5% of patients, and the most common medications were antiviral (91.4%). Results revealed that except oxygen therapy, other treatment methods for COVID-19 significantly correlate with mortality (Table-4).

### Role of Complications of COVID-19 on the Mortality Rate

Regarding Table-5, AKI, the most common complication of COVID-19, was observed in 33 patients. Also, the chi-square test indicated that complications of COVID-19 were significantly correlated with mortality (Table-5). In addition, logistic regression showed that shock (OR: 69.67, 95%CI: 10.35 to 468.78, P˂0.001) was the most important complication of COVID-19 that was significantly related to mortality.

## Discussion

COVID-19 has severely affected the health system of countries worldwide. People with chronic disorders, including DM patients, are more susceptible to COVID-19 in comparison with other people [7]. Evidence also indicates that DM is among the most frequent comorbidities in COVID-19 cases that are hospitalized in the ICU. In general, when individuals with DM suffer from a viral infection, treatment is more difficult due to changes in BS levels and possibly the presence of its complications. Also, in patients with DM, the immune system is compromised, which makes the disease more difficult and can prolong the recovery period [8]. DM can increase the risk of immune system disorders, and evidences demonstrated that DM could interrupt immunity by reducing the functions of the immune system through the disruption of the chemotaxis of neutrophils, the antibacterial activity of monocytes, and phagocytosis, which lead to an increase in infection [9].

In research by Richardson *et al*. it was determined that the most common chronic diseases in COVID-19 cases are HTN (56%), obesity (41%), and DM (33%) [10]. On the other hand, Zhou *et al*. [3] stated that 19% of people with COVID-19 have DM. Therefore, it is important and necessary to check the condition of DM among patients with COVID-19 [3].

Al-Salameh *et al*. indicated that the mean age of the cases was 72 years, and 55% of the patients were male [11]. A great number of deaths were reported in non-ICU units and elderly patients [11]. Also, DM was not correlated with death but was correlated with admission to the ICU and duration of hospitalization. Age was positively correlated with being admitted to ICU and mortality. A quarter of cases hospitalized with COVID-19 had DM, and DM was related to a higher risk of admission to the ICU but not to a significant increase in mortality [11]. In our study, in line with Al-Salameh *et al*. [11] findings, most of the deaths occurred in elderly patients hospitalized in the ICU, and in contrast to their study, the rate of mortality in DM cases who were infected with COVID-19 was significantly higher. Indeed, DM cases, especially cases with type 2 DM, need more special care due to the occurrence of multiple complications and older age, and of course, with more complications, the cases leading to death are also increased [12]. During the COVID-19 pandemic, DM cases are at high risk of requiring ICU admission, and managing DM in the ICU is always demanding. However, when DM cases develop COVID-19, the situation becomes more complicated [13]. In DM cases infected with COVID-19, the exact risk factors for ICU admission are unclear. However, glycemic management in DM cases is observed to be a crucial predictor of any type of infection.

In the study by Sheng *et al*. [14], it was noted that shock was frequent (5 to 10%) in cases with COVID-19. Also, in critical patients under intensive care, its prevalence reaches 67% and is associated with high mortality rates [14]. In our study, the shock frequency in DM cases with COVID-19 was 11%, and this rate reached 25% in individuals admitted to the ICU. A high rate of death (87%) has been observed in patients with shock complications [14]. It shows the importance of shock management in DM cases with COVID-19. Optimal management requires rapid identification with accurate assessment and differentiation. Correction of hypoperfusion and treatment of the underlying process are essential aspects of treatment.

On the other hand, in our study, AKI was seen in 15.6% of patients, which was correlated with high mortality, so in patients who suffered from AKI, the mortality rate was 43%, which was 8-fold higher than that of other patients without AKI. In Khalili *et al.* study [15], 58 cases (22.8%) had AKI, and DM cases were at a higher risk of developing AKI and also suffered from more severe kidney injuries compared with non-diabetic cases. The frequency of AKI amongst cases of COVID-19 varied widely and ranged from zero to 37% [15]. The differences in the measurement of serum creatinine levels and the definitions of AKI in different studies may cause differences in the rate of AKI. A probable alternative reason for the broad spectrum of AKI frequency may be the distinct characteristics and disease severities in the studied cases. In our research, patients were referred to an academic hospital in the center of the Semnan province, which generally accepts COVID-19 cases with higher disease severity. Some studies in American and European centers have reported a frequency of 20-40% for AKI in COVID-19 cases, while early reports from China indicated that a smaller percentage of cases with COVID-19 develop AKI [16][17].

In our study, the need for hospitalization in the ICU was 43%, the mortality rate was 21%, and the mortality rate increased significantly and reached 48% in cases admitted to the ICU. The most important underlying disease in our study was HTN that observed in 60% of patients.

In the study by Roncon *et al*. [18], the primary outcome in COVID-19 cases with DM was the possibility of ICU admission, and the secondary outcome was the risk of mortality in all DM cases with COVID-19. In their study, which was similar to our study in terms of gender distribution (57% men), DM was the second most frequent underlying disease. Also, patients with DM showed a considerable increase in the risk of admission to the ICU and were at further risk of mortality [18].

Several factors, such as aging, obesity, cardiovascular diseases, and stroke are correlated with a higher risk of death due to COVID-19 in patients with DM [19]. Actually, patients with DM infected with COVID-19 demonstrate severe clinical problems, increased ICU hospitalization, need for mechanical ventilation, and a significant increase in inflammatory markers. Indeed, DM as a risk factor for COVID-19-related mortality should be considered as the priority of treatment [20][21].

## Conclusion

According to the obtained results, the presence of underlying diseases, including DM, could increase the probability of contracting COVID-19 and its related mortality rate. Therefore, treating COVID-19 in patients with DM requires an integrated team approach to minimize the possibility of complications and mortality. In addition, preventive strategies should be adopted to care for individuals with comorbidities. Also, these strategies help to reduce both complications and mortality rates amongst patients, as well as related financial burdens on the healthcare system.

## Conflict of Interest

There are no any conflicts of interest.
